# Larval taste exposure creates compound-specific behavioral modifications in adult *Drosophila melanogaster*

**DOI:** 10.1016/j.isci.2026.117467

**Published:** 2026-09-08

**Authors:** Vaibhav D. Menon, Christopher Creighton, Mayra Vazquez-Muñoz, Anupama Dahanukar

**Affiliations:** 1 Department of Molecular, Cell & Systems Biology, University of California, Riverside, Riverside, CA 92521, USA; 2 Interdepartmental Neuroscience Graduate Program, University of California, Riverside, Riverside, CA 92521, USA; 3 Division of Biomedical Sciences, University of California, Riverside, Riverside, CA 92521, USA

**Keywords:** neuroscience, *Drosophila*, taste, chemosensation, gustatory receptor, critical period, bitter taste, prior experience

## Abstract

An animal’s ability to taste is critical for nutrient selection and rejection of potentially toxic substances. Factors such as age, diet, environment, and internal state can also influence food-related behavioral outcomes. One such factor—exposure to taste cues during early development—remains poorly understood. Here, we evaluate how larval taste input affects adult feeding preference in *Drosophila melanogaster*. We find that larval dietary exposure to papaverine, a bitter alkaloid, attenuates feeding avoidance of papaverine in adult flies. This behavioral modification appears compound specific, occurring with papaverine but not with other bitter chemicals tested, and selective, with feeding preferences for other bitter tastants unaffected. We identify contributions of larval bitter taste function, taste projection populations, and the cyclic AMP (cAMP)-phosphodiesterase dunce in supporting this behavioral modification. Together, our findings suggest that developmental experiences with bitter compounds can change taste-mediated behaviors through defined sensory and neural circuit components.

## Highlights

• Larval papaverine exposure attenuates adult papaverine avoidance
• Papaverine-induced plasticity is compound- and taste organ-specific
• Adult taste changes require larval bitter sensing and mlSEZt neurons
• cAMP-dependent memory pathways support trans-metamorphic taste plasticity

## Graphical abstract

**Figure:**
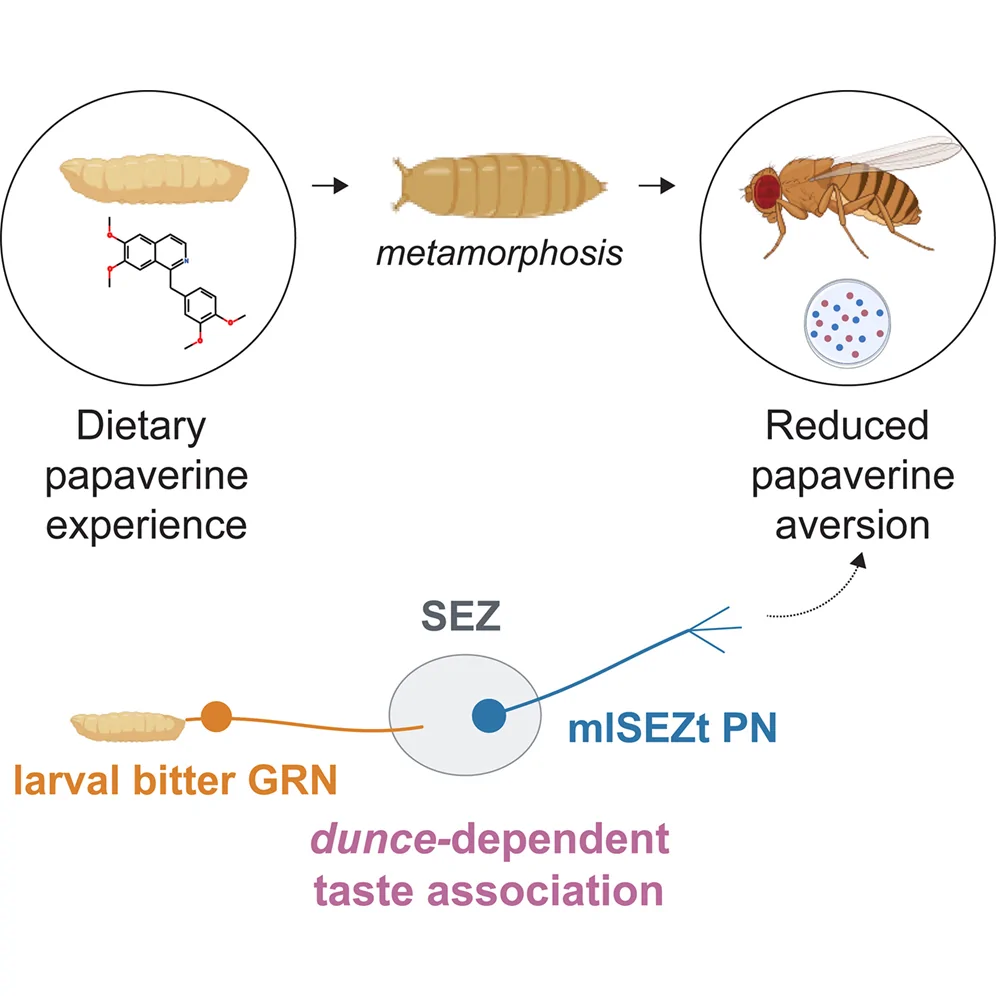

## Teaser

Behavioral neuroscience; Biological sciences; Developmental biology; Neuroscience

## Introduction

Across species, taste guides essential feeding decisions by promoting the ingestion of nutrients and avoidance of toxins. Although innate taste responses are well characterized, taste perception is highly plastic, adapting to environmental changes, nutritional status, and prior sensory experience. Taste perception dynamically adjusts to nutrient availability, optimizing dietary intake to maintain homeostasis. Importantly, developmental stage influences an organism’s capacity for sensory plasticity: during early life, animals rapidly incorporate environmental information through robust neural and anatomical changes. However, the specific pathways through which early gustatory experience may shape adult feeding behavior remain unclear.

Early-life sensory experiences have long-lasting influences on adult feeding preferences. Studies in mammals demonstrate that exposure to flavors during prenatal or early postnatal stages can profoundly shape dietary choices later in life,^1^^,^^2^^,^^3^^,^^4^^,^^5^ with recent work showing that exposing weaning mice to sucrose perturbs developing inhibitory circuits in the insular cortex to enhance sucrose preference in a prolonged manner.^6^ Similarly, insect larvae exposed to certain chemical cues exhibit modified adult behaviors, with extensive evidence from studies of olfactory conditioning.^7^^,^^8^^,^^9^ In contrast, taste-driven conditioning of adult behavior by larval experience is less characterized. Several moth species shift oviposition preference on food substrates based upon larval experience,^8^^,^^10^^,^^11^ positioning taste as a potential avenue to influence adult behavior. However, the mechanisms by which observed behavioral changes persist across the dramatic reorganization of metamorphosis remain poorly understood.

Recent studies have revealed sophisticated mechanisms underlying trans-metamorphic behavioral persistence. Although most larval neural connections are lost during metamorphosis, behavioral information can persist through three distinct strategies: neural remodeling for continued function, trans-differentiation to serve alternative circuits, and anatomical reorganization during pupal development.^12^ Critically, this work established that trans-metamorphic behavioral effects do not require direct neural persistence but can emerge through developmental programs that reconstruct functionally equivalent circuits. Complementing this framework, the finding that larval microbiota exposure programs adult gustatory responses through stage-specific requirements for *Gr66a*^+^ neuron function provided direct evidence for critical period effects in taste development.^13^ The findings revealed that the first 48–72 h of larval development constitute a critical window for taste circuit programming, with germ-free flies losing gustatory responses to bacterial peptidoglycan entirely.

Larval taste experiences may also influence adult behavior through epigenetic mechanisms that create stable molecular memory across metamorphosis. Recent work has shown that larval nutritional conditions induce lasting changes in adult dopamine signaling through histone modifications in mushroom bodies,^14^ while G9a-mediated histone methylation regulates behavioral differences through chromatin remodeling.^15^ Such epigenetic pathways could provide molecular substrates for taste memory that survive the extensive neural reorganization of metamorphosis.

The vinegar fly, *Drosophila melanogaster*, provides a versatile model to address these questions. The fly’s gustatory system includes specialized appetitive (e.g., sugar-sensing) or deterrent (e.g., bitter-sensing) gustatory receptor neurons (GRNs) that respond robustly to various classes of tastants and has been extensively characterized at both the behavioral and molecular level over the past quarter century.^16^^,^^17^ In adult flies, associative taste learning engages well-defined central circuits including dopaminergic inputs into the mushroom body.^18^ Other forms of taste plasticity can arise from peripheral sensory neuron adaptation and habituation, which may involve higher order neuronal circuits. Recent connectomic analyses have revealed that taste circuits are organized with largely modality-specific segregation at second-order neurons but integration across modalities at third-order levels,^19^^,^^20^^,^^21^ providing anatomical frameworks for understanding how early taste experiences could influence adult circuit function.

Despite the dramatic anatomical and nervous system-wide reorganization during metamorphosis in holometabolous insects like *D*. *melanogaster*, food-related sensory experiences in larvae can shape adult behaviors. Conditioning of behavioral responses following larval exposure has been observed in *Drosophila*,^22^^,^^23^^,^^24^ with prior work demonstrating a requisite role for larval bitter sensing in adult avoidance of peptidoglycan.^13^ However, several critical questions remain unresolved regarding the mechanisms and specificity of such trans-metamorphic effects. For instance, does larval exposure to aversive compounds always increase adult avoidance, or can it lead to attenuation through habituation mechanisms? Is the effect of larval exposure generalizable across aversive chemicals, or do compound-specific effects reflect specialized neural circuits or molecular mechanisms? How do downstream components of bitter circuits control the response to re-exposure?

The notion that taste information may persist across metamorphosis is particularly intriguing given that most larval GRNs, except for pharyngeal GRNs, do not survive metamorphosis.^25^ Additionally, vast mushroom body reorganization during metamorphosis^12^ suggests that any persistent anatomical trace would require sophisticated molecular mechanisms to maintain functional relevance. Ecological considerations further support the biological relevance of developmental taste plasticity. In natural environments, larvae spend nearly their entire developmental period immersed in nutritive environments rich in taste cues, making the study of how gustatory experiences influence long-term feeding decisions both ecologically and neurobiologically important.

Here, we leverage *Drosophila melanogaster* to investigate whether and how larval exposure to aversive tastants alters adult feeding behavior. We established a developmental exposure paradigm using three bitter compounds and employed multiple behavioral assays to characterize adult responses. Using genetic approaches including mutant analysis, targeted neuronal silencing, and temporal control systems, we examined the neural circuit requirements for behavioral modification. Our key findings demonstrate that larval exposure to papaverine (PAP) specifically attenuates adult PAP avoidance in a compound-specific and taste organ-specific manner that persists for at least 2 weeks post exposure. Genetic dissection experiments reveal requirements for *Gr33a*^+^ bitter GRNs during larval development, bitter projection neurons (mlSEZt, mediolateral SEZ tract), and cyclic AMP (cAMP) signaling components (*dunce* and *amnesiac*), as well as bitter-sweet integration pathways involving Obp49a (odorant-binding protein 49a) and GABA (gamma-aminobutyric acid) signaling. Single-sensillum recordings (SSRs) further suggest that larval PAP exposure could weakly disinhibit sweet neuron responses. Notably, exposure to alternative bitter compounds (noscapine [NOS] and denatonium [DEN]) failed to generate self-attenuation and instead enhanced adult PAP avoidance, revealing unexpected cross-compound interactions. Together, these findings indicate that specific early gustatory experiences can produce lasting behavioral modifications in tastant-specific ways, and they establish a tractable model for investigating the precise cellular and molecular basis of trans-metamorphic information transfer of taste experience.

## Results

### Larval exposure to bitter tastants attenuates avoidance in a context-specific manner

**Figure 1:**
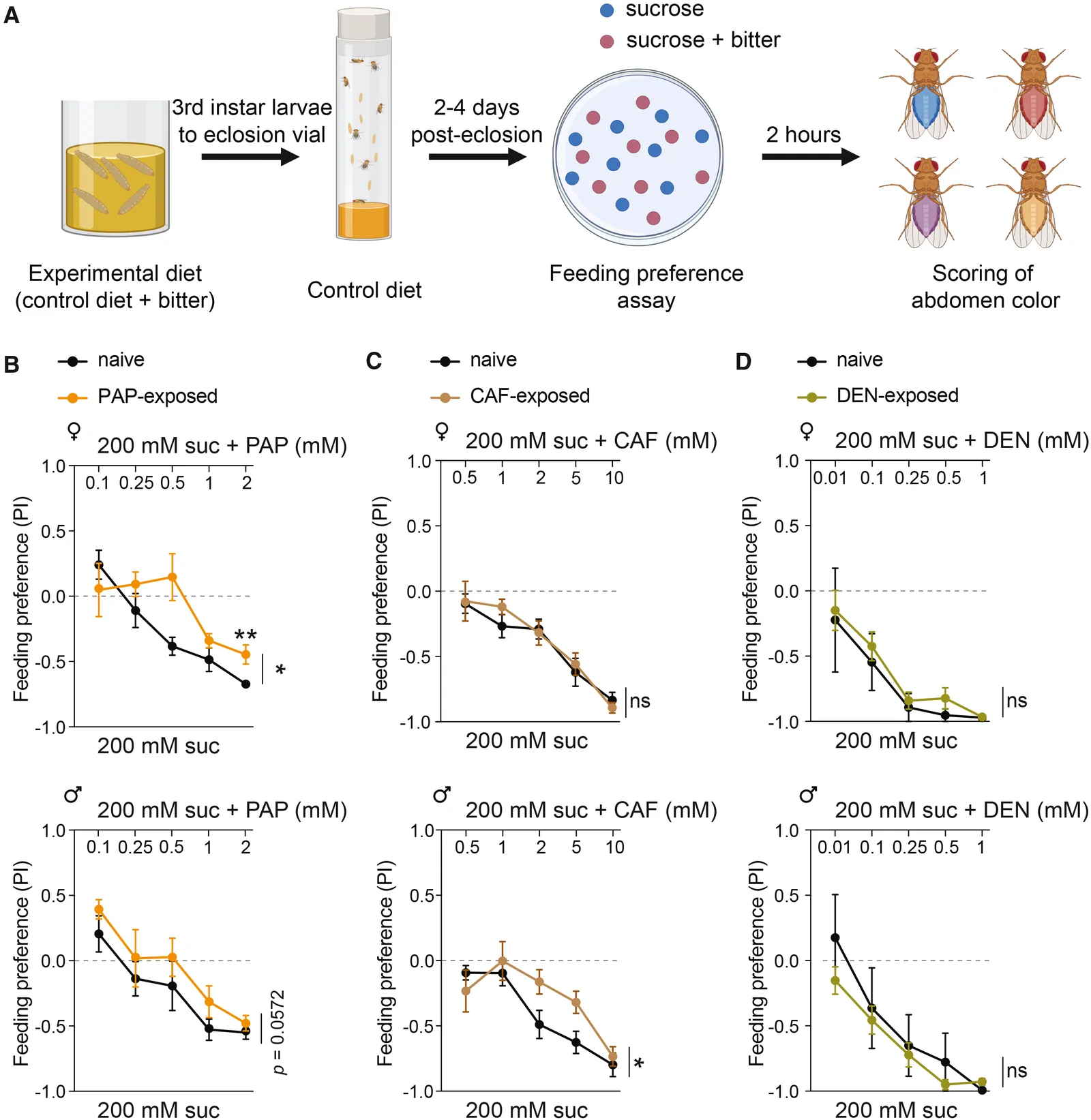
Larval exposure drives attenuation of avoidance in a tastant, dose, and sex-specific manner (A) Schematic of rearing and experimental pipeline, including exposure model. Larvae were reared in experimental diet until late 3rd instar/pupariation, rinsed, and transferred to eclosion chambers (vials containing control diet). 2–4 days post-eclosion, starved flies were fed in binary choice arenas for 2 h. Gut dissection was performed to score food preference. (B–D) Feeding preference (PI, preference index) of females (top) and males (bottom) choosing between 200 mM sucrose and 200 mM sucrose mixed with indicated concentrations of (B) papaverine (PAP; ♀ [*n* = 4–46], ♂ [*n* = 3–45]), (C) caffeine (CAF; ♀ [*n* = 10–12], ♂ [*n* = 11–12]), or (D) denatonium [DEN; ♀ [*n* = 3–12], ♂ [*n* = 3–13]). Bars and error bars represent mean ± SEM. All dose-response curves were analyzed via two-way ANOVA followed by Šídák’s multiple-comparisons test. Statistical test of rearing effect is indicated to the right of each graph; asterisks at specific doses indicate significant pairwise differences between naive and exposed groups. ∗*p* < 0.05 and ∗∗*p* < 0.01; ns = not significant (*p* > 0.05).

We examined the effect of larval exposure to select bitter tastants using three chemicals that represent a spectrum of avoidance responses in previously reported choice assays.^26^ With this approach, we asked how wild-type flies reared on media containing caffeine (CAF), PAP, or DEN would behave as adults when presented with these compounds. These compounds previously elicited low, moderate, and high levels of avoidance, respectively, in feeding choice assays.^26^ As larvae reached the wandering third instar stage or initiated pupariation, we collected and washed them before transferring to vials containing standard food media. Our control condition, termed “naive,” involved exposure to standard media throughout development, with identical handling, including washing of late instar larvae and early pupae prior to transfer to standard food-containing eclosion vials (see STAR Methods) (Figure 1A).

We performed dose response experiments in 2- to 4-day-old adults, offering flies a choice between 200 mM sucrose and 200 mM sucrose + a bitter compound. We selected this choice paradigm because bitter chemicals are detected at low concentrations; we predicted that shifts in behavior might be obscured by high concentrations that could generate absolute avoidance regardless of rearing history. Across the dose-response panel, PAP-reared female flies exhibited reduced avoidance of the sucrose-PAP mixture compared to naive controls, while a similar but non-significant trend was observed for males (Figure 1B). Notably, female PAP-reared flies reacted to 2 mM PAP with similar preference as naive flies showed to 0.5 mM PAP, suggesting approximately a 4-fold shift in response across this concentration range (Figure 1B). Rearing larvae on CAF media resulted in reduced CAF sensitivity in adult males but not females (Figure 1C). Larval DEN exposure did not produce detectable sensitivity shifts in either sex (Figure 1D). Overall, the effect of larval exposure appears compound and dose specific, with potential concentration-dependent differences between sexes.

### Persistent sensitivity shifts arise independently of developmental toxicity or compound perdurance

**Figure 2:**
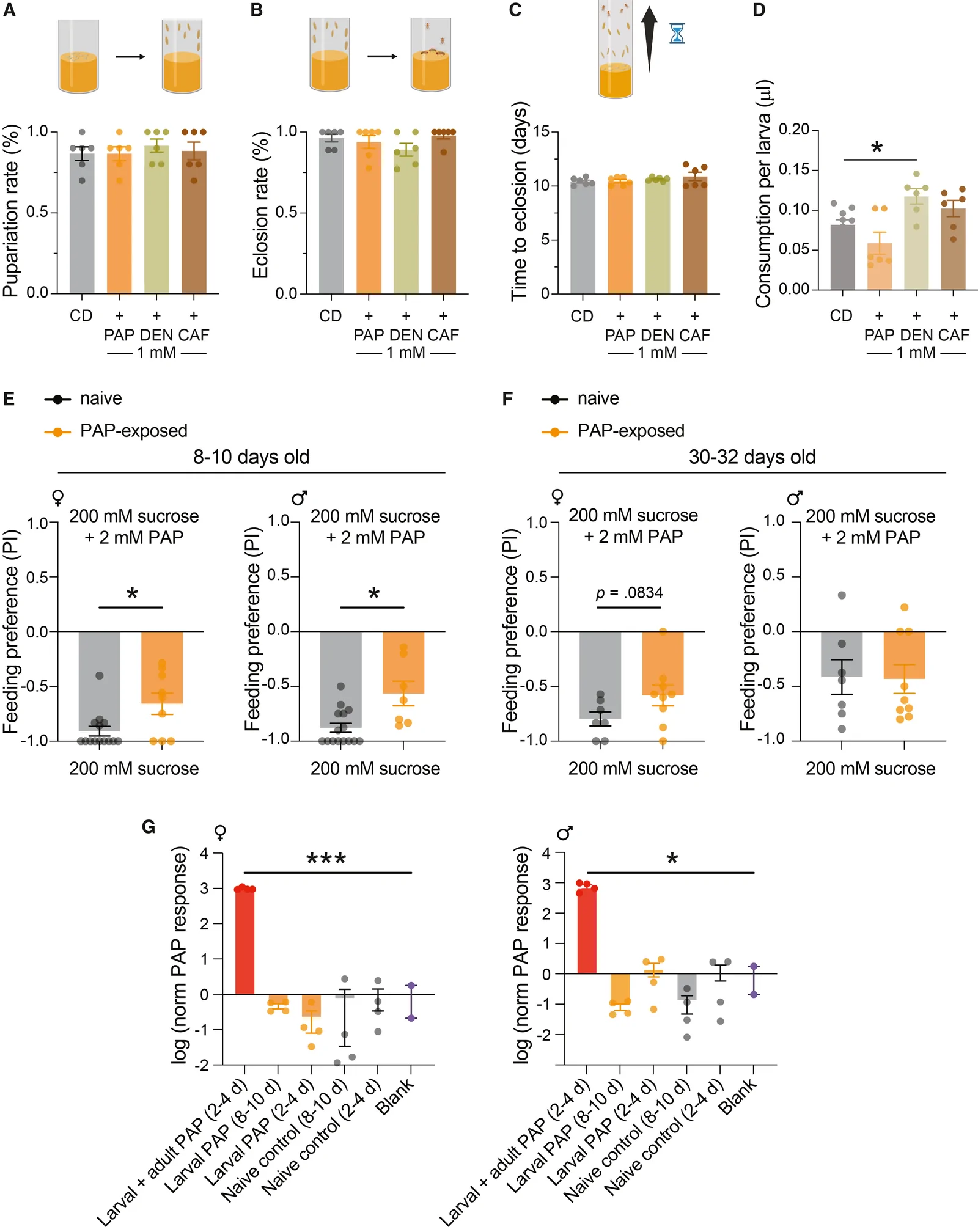
Bitter rearing does not cause toxicity or elicit significant chemical perdurance across metamorphosis (A–D) Mean rates of (A) pupariation, (B) eclosion, (C) development time, and (D) larval food consumption (*n* = 6–10) for flies reared on control diet (CD) or diets supplemented with 1 mM papaverine (PAP), denatonium (DEN), or caffeine (CAF). (E and F) Feeding preference (PI, preference index) of flies aged (E) 8–10 days (*n* = 7–15) or (F) 30–32 days (*n* = 7–9), choosing between 200 mM sucrose and 200 mM sucrose mixed with 2 mM PAP. Flies were given CD (naive) or CD with 1 mM PAP (PAP-exposed) as larvae. (G) Mean normalized PAP detection peaks from whole-body female (left) and male (right) tissues via mass spectrometry (*n* = 2–4); peaks identified by PAP standard retention times. All bars represent mean ± SEM. Developmental, consumption, and mass spectrometry data (A–D and G) were analyzed via Welch’s ANOVA with Dunnett’s T3 multiple-comparisons test. Behavioral data (E and F) were analyzed via Welch’s *t* test. ∗*p* < 0.05 and ∗∗∗*p* < 0.001.

We investigated whether the observed behavioral sensitivity shifts reflected physiological consequences of potential toxicity from bitter ingestion during development. Larvae exposed to PAP, DEN, and CAF showed pupariation rates equivalent to control larvae (Figure 2A), indicating normal initiation of metamorphosis. Bitter-reared larvae also showed adult eclosion frequencies similar to those reared on control diet (Figure 2B), and the time from egg hatching to eclosion was not significantly delayed across the tested diets (Figure 2C). Because rearing in a bitter environment could in principle modulate larval feeding behavior, we next asked whether larval food intake itself was disrupted by a nutritive environment containing bitter chemicals using a colorimetric consumption assay (Figure 2D). We found that the intake of PAP- and CAF-laced food did not differ from the control diet, although that of DEN-laced food was slightly elevated. Together, these results suggest that our bitter rearing conditions do not adversely affect survival, developmental timing, or larval food intake.

Given that feeding behavior in *Drosophila* modulates with age as metabolic costs and energetic demands change,^27^^,^^28^ we next tested the persistence of these sensitivity shifts over time. We tested 8- to 10-day-old adults reared as larvae on PAP-treated food in binary choice experiments, comparing preference for 200 mM sucrose versus 200 mM sucrose + 2 mM PAP. PAP-exposed flies, at this point 12–14 days since their last PAP encounter, still avoided the sucrose/PAP substrate significantly less than naive, age-matched counterparts (Figure 2E). PAP-reared females aged 30–32 days continued to exhibit weak attenuation of PAP avoidance, although this did not reach statistical significance (Figure 2F). Thus, the effect of larval PAP exposure persists into adulthood, but may be reversed over time as described for taste-associated memory in previous studies.^27^^,^^28^

A previously proposed “chemical legacy” hypothesis posits that trace amounts of chemicals from the larval environment might persist within the insect body throughout metamorphosis and condition behavioral responses.^29^^,^^30^ In principle, our rearing and handling protocol addresses external chemical residue through thorough rinsing of collected larvae and pupae. However, we could not exclude the possibility that trace amounts of chemical ingested by the animal might influence adult behavior. Mass spectrometry analysis of whole adult bodies revealed no detectable PAP-associated peaks in PAP-exposed female or male flies compared to sample blanks (Figure 2G). In contrast, flies continuously exposed to PAP during both larval and adult stages showed significantly higher detection peaks than both PAP-reared and naive flies. These findings suggest that PAP rearing-induced behavioral shifts are unlikely to result from residual chemicals present in adult tissues, although we acknowledge the detection limits of our analytical approach.

### Larval bitter exposure affects behavioral sensitivity in a taste organ-specific manner

**Figure 3:**
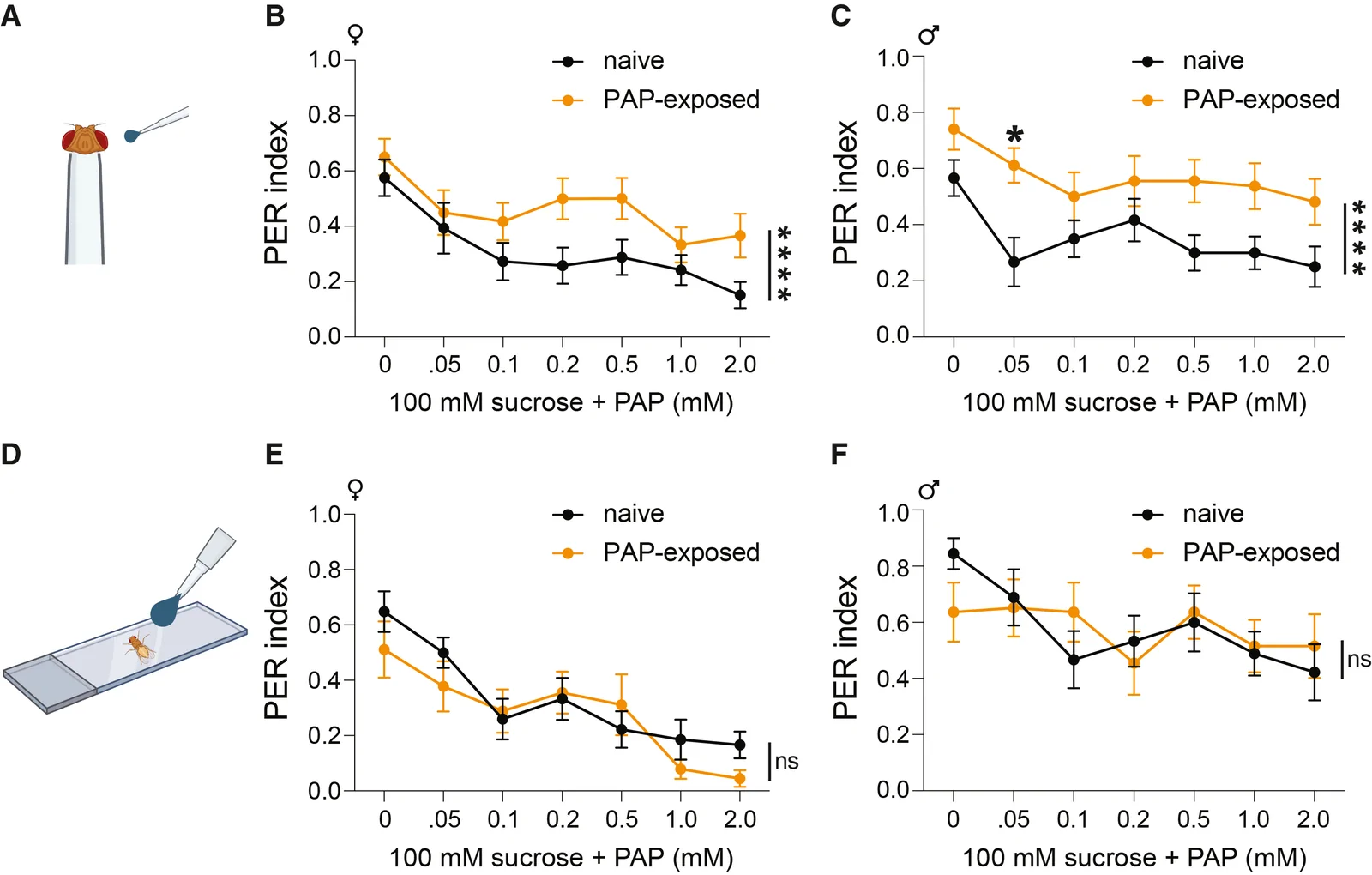
Larval PAP exposure drives bitter desensitization response in proboscis extension reflex response assays (A) Schematic of the labellar proboscis extension response (PER) assay, in which stimuli are presented to the labellum of a fly immobilized inside a pipette tip. (B and C) PER index of adult females (B) and males (C) stimulated with the indicated tastants (*n* = 14–22). (D) Schematic of the tarsal PER assay, in which stimuli are presented to the tarsi of a fly immobilized on a microscope slide by the dorsal side of its thorax. (E and F) PER index of adult females (E) and males (F) stimulated with the indicated tastants (*n* = 11–18). Flies were given control diet (naive) or control diet with 1 mM PAP (PAP-exposed) as larvae. Data points represent mean PER frequency ±SEM (per group). PER data were analyzed via two-way ANOVA followed by Šídák’s multiple-comparisons test. Statistical test of rearing effect is indicated to the right of each graph; asterisks at specific doses indicate significant pairwise differences (∗*p* < 0.05 and ∗∗∗∗*p* < 0.0001). ns = not significant (*p* > 0.05).

Multiple studies have demonstrated that flies diminish their proboscis extension response (PER), a sign of taste acceptance, and retract their proboscis in response to aversive tastants.^31^^,^^32^^,^^33^ To better understand how larval bitter exposure affects adult feeding behavior, we asked if PAP-reared flies exhibit modified PER responses upon labellar stimulation (Figure 3A). We tested sucrose mixed with PAP concentrations from 0 mM to 2 mM PAP, presenting stimuli in random order to prevent response conditioning. In both females and males, PAP-reared flies demonstrated significantly higher PER frequencies than naive flies upon stimulation of the labellum (ANOVA *p* [rearing] < 0.0001) (Figures 3B and 3C).

Organ-specific projection patterns of taste information from different peripheral organs suggest dedicated processing circuits for different input sources.^34^^,^^35^^,^^36^^,^^37^ To determine whether changes in behavioral bitter sensitivity were specific to labellar taste circuits, we conducted tarsal-evoked PER experiments, assessing proboscis extension in response to stimulation of tarsal sensilla rather than labellar sensilla (Figure 3D). When we stimulated tarsi, PAP-reared flies did not differ from naive flies in their PER (Figures 3E and 3F). This finding is consistent with the idea that larval PAP exposure generates organ-specific shifts in taste sensitivity.

### Adult food interactions are modified by larval experience

**Figure 4:**
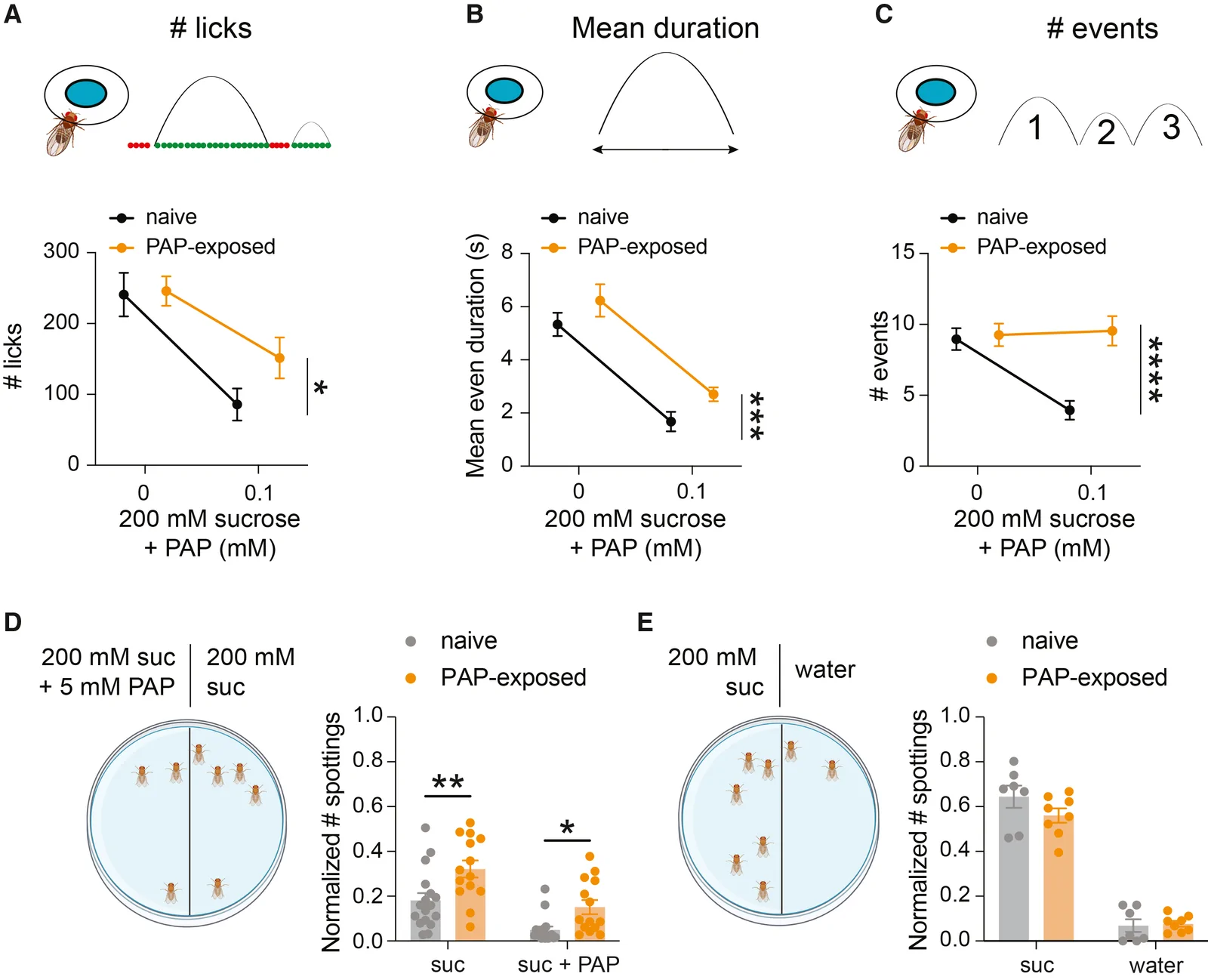
Adult approach of PAP-laced food is enhanced by larval PAP exposure (A–C) Micro-feeding parameters of adult females given 200 mM sucrose alone or mixed with 0.1 mM PAP in FLIC assays. Graphs show (A) total number of licks, (B) mean interaction event duration, and (C) total number of interaction events per fly (*n* = 47–103). (D and E) Normalized distribution (spottings) of flies recorded every 15 min over 2 h on indicated food surfaces (*n* = 7–17). Flies were given control diet (naive) or control diet with 1 mM PAP (PAP-exposed) as larvae. All data show mean ± SEM. Interaction data (A–C) were analyzed via Mann-Whitney tests. Residence data (D, E) were analyzed via two-way ANOVA with Šídák’s multiple-comparisons test. Asterisks indicate significant differences between naive and exposed groups (∗*p* < 0.05, ∗∗*p* < 0.01, ∗∗∗*p* < 0.001, and ∗∗∗∗*p* < 0.0001).

Given the labellum-specific modification in taste sensitivity observed in PER experiments, we asked whether larval PAP experience influenced feeding behavioral features linked to labellar usage. Using the fly liquid-food interaction counter (FLIC), we measured the duration and frequency of feeding interactions with food sources.^38^ We tested PAP-reared or naive female flies with either 200 mM sucrose or 200 mM sucrose + 0.1 mM PAP. PAP-reared flies interacted with the sucrose-PAP solution more frequently and showed a trend toward longer interaction compared to naive flies (Figures 4A–4C), suggesting increased chemosensory-mediated approach behavior.

Previous work has shown that taste input contributes to preference for substrate residence when flies are given choices between different substrates.^39^ Thus, we asked if the behavioral sensitivity shift observed in PAP-exposed flies altered residence behavior. Populations of female flies were given two agarose-based substrates containing sucrose or a sucrose-PAP mixture, and the number of flies residing on each substrate was counted every 15 min for 2 h. When presented with sucrose and sucrose + PAP substrates, PAP-reared flies showed more total spottings on both substrates compared to naive flies (Figure 4D), indicating increased approach/residence behavior in environments containing PAP. Importantly, this effect was not observed when flies were tested with sucrose versus water (Figure 4E), showing that PAP exposure does not broadly increase activity or sucrose-driven residence. In environments where PAP is available, PAP-reared flies may demonstrate more robust chemosensory-mediated approach behaviors, possibly through a reduction in behavioral sensitivity to PAP, although the specificity of environmental influence warrants further investigation.

### Bitter exposure-driven modifications show tastant specificity

**Figure 5:**
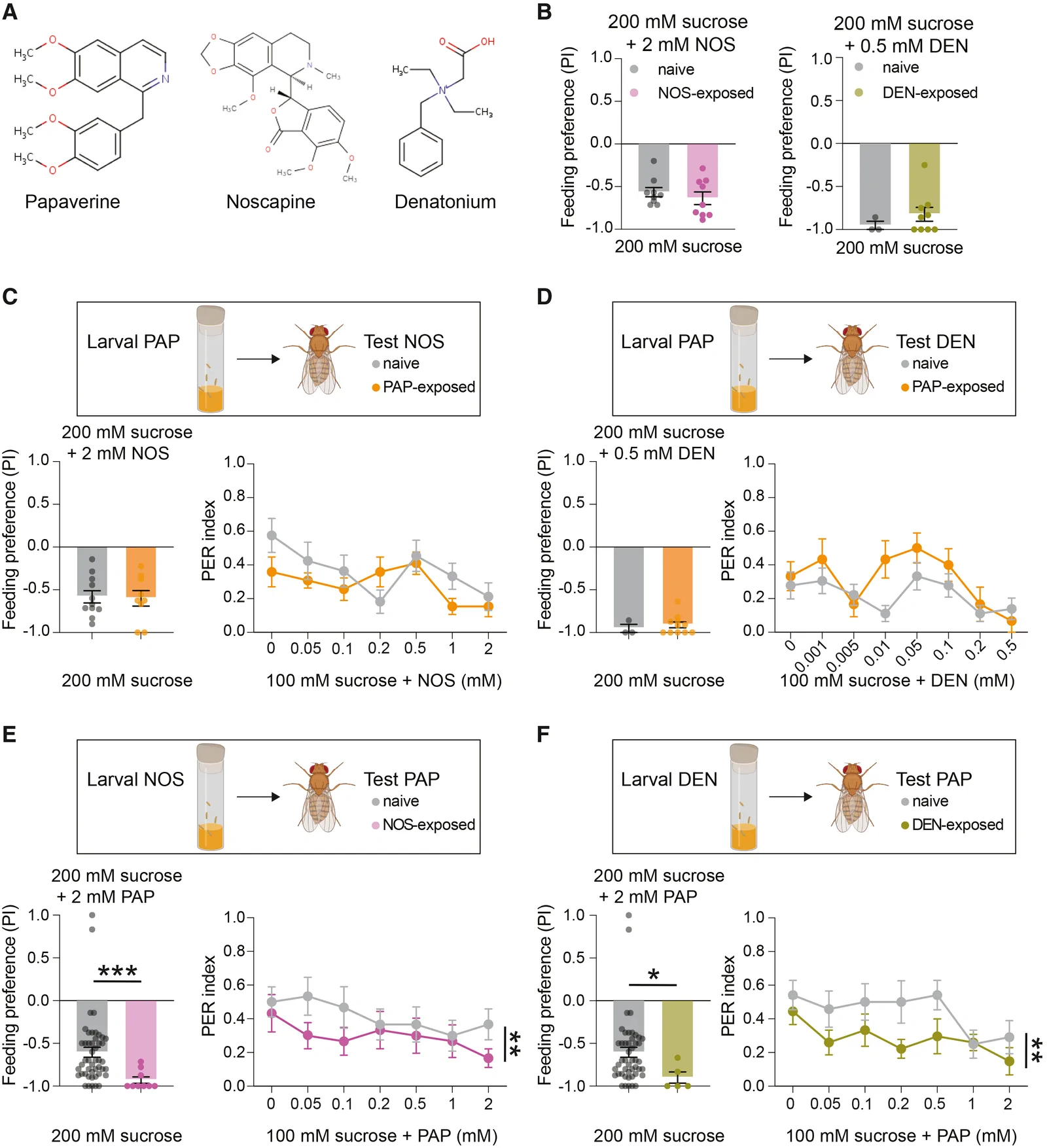
Effects of larval bitter-tastant exposure on adult feeding responses are asymmetric and tastant dependent (A) Chemical structures of papaverine (PAP), noscapine (NOS), and denatonium (DEN). PAP and NOS share a benzylisoquinoline backbone. (B) Feeding preference (PI, preference index) of flies given control diet (naive) or control diet with 2 mM NOS (NOS-exposed) or 0.5 mM DEN (DEN-exposed) as larvae; flies were tested in binary choice assays with 200 mM sucrose versus 200 mM sucrose + 2 mM NOS (*n* = 9–45) (left) or 200 mM sucrose + 0.5 mM DEN (*n* = 3–9) (right). (C–F) Feeding preference (PI, preference index) and proboscis response (PER index) of flies subjected to larval cross-exposure treatments as shown and tested with NOS, DEN, or PAP as indicated. All data show mean ± SEM. *n* = 3–47 for PI and *n* = 8–11 for PER index. PI comparisons were analyzed using Welch’s *t* test or the Mann-Whitney test depending on normality. PER dose-response curves were analyzed using two-way ANOVA with Šídák’s multiple-comparisons test where applicable. ∗*p* < 0.05, ∗∗*p* < 0.01, and ∗∗∗*p* < 0.001.

Heterogeneity in bitter taste responses has been observed in *Drosophila*, suggesting that different bitter compounds may recruit distinct behavioral mechanisms.^33^ Does early exposure to PAP confer attenuated responses to other bitter compounds? Conversely, is exposure to any bitter compound sufficient to reduce avoidance to PAP? To address these questions, we reared flies on media containing one of three bitter chemicals: PAP, NOS, or DEN. NOS and PAP are both benzylisoquinoline alkaloids, sharing a backbone typical of poppy-derived compounds, whereas DEN is a synthetically derived cation (Figure 5A).^33^

Like PAP, DEN and NOS elicited robust avoidance in naive flies, but unlike PAP, larval exposure to neither DEN nor NOS reduced adult avoidance of the respective compound in choice assays (Figure 5B). To test how larval PAP exposure affected adult responses to other bitter tastants, we reared larvae on PAP and tested their adult responses to NOS in choice and PER assays. PAP-exposed flies phenocopied their naive counterparts exhibiting strong aversion to the sucrose + NOS mixture in choice assays and similar PER responses to sucrose + NOS solutions (Figure 5C). We next probed the consequence of PAP exposure on adult responses to a structurally dissimilar bitter, DEN, and again observed no clear effect of larval PAP experience on adult responses to DEN in either choice or PER assays (Figure 5D).

Conversely, when we tested adult PAP responses in flies exposed to NOS as larvae, we observed increased PAP avoidance compared to naive flies in feeding choice assays (Figure 5E). In PER analysis of responses to the sucrose + PAP panel, linear regression analysis of the naive vs. NOS-exposed curves revealed a significantly lower elevation, or intercept, for NOS-exposed flies, consistent with the increased avoidance in binary choice assays (Figure 5E). DEN-reared flies showed a similar shift in avoidance of PAP (Figure 5F) and parallel reductions in PER to sucrose + PAP solutions compared to naive flies (Figure 5F). Together, these results uncover two compound-specific findings: first, adult attenuation of PAP avoidance requires larval PAP exposure specifically, and second, larval exposure to alternative bitter compounds appears to enhance rather than reduce adult PAP avoidance.

### Larval exposure-dependent behavioral changes involve taste pathways

**Figure 6:**
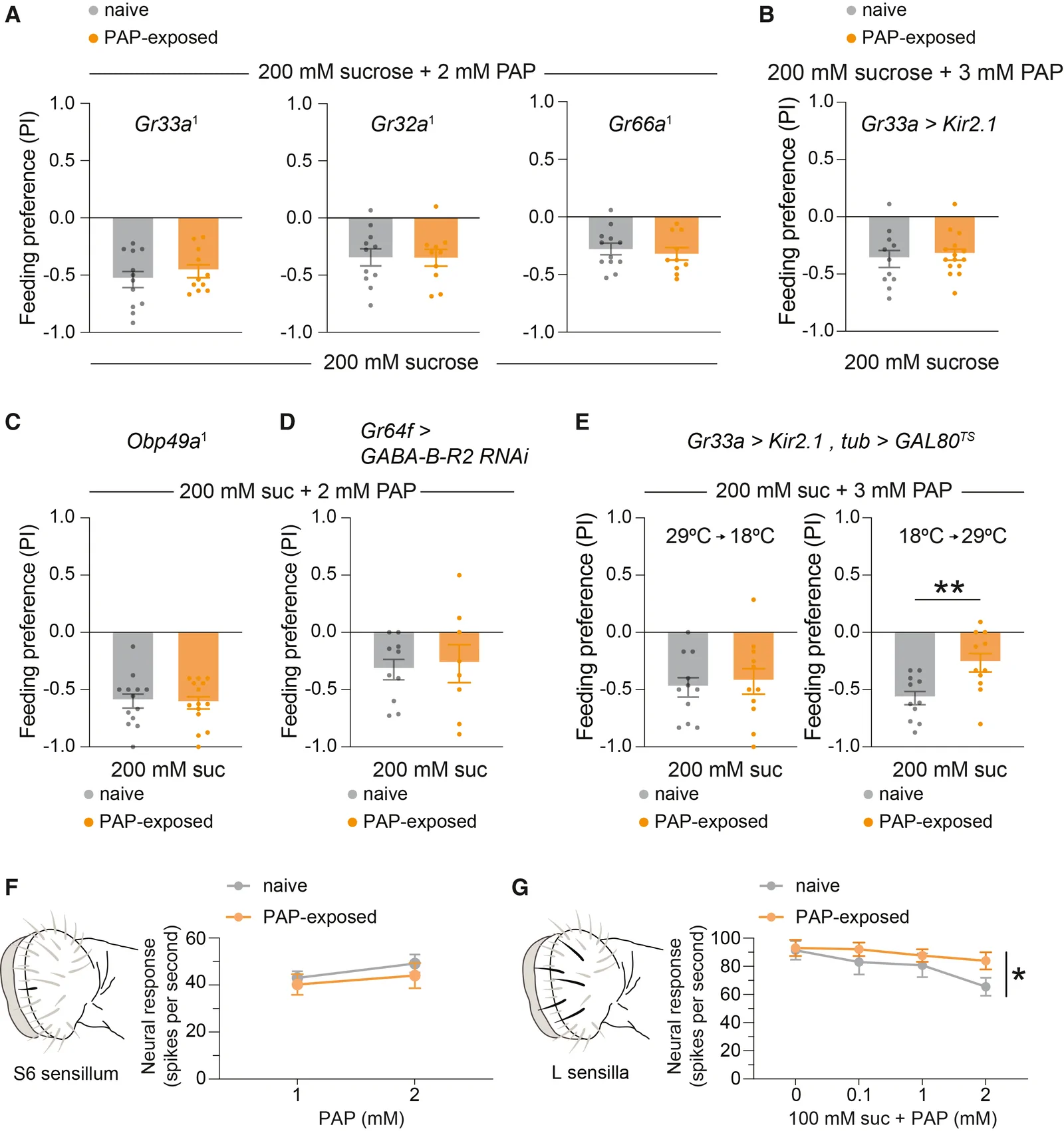
Bitter neuron activity and bitter taste detection are required for effect of larval exposure (A and B) (A) Feeding preference (PI, preference index) of *Gr33a*^1^*/Gr33a*^1^; *+/+* (*Gr33a*) and Gr32a^MI04430^*/Gr32a*^MI04430^; *+/+* (*Gr32a*) and *Gr66a*^1^*/Gr66a*^1^; *+/+* (*Gr66a*) mutants choosing between 200 mM sucrose and 200 mM sucrose mixed with 2 mM papaverine (PAP). (*n* = 10–12) (B) Feeding preference (PI) of *UAS-Kir2*.*1/+*; *Gr33a-GAL4/+* flies choosing between 200 mM sucrose and 200 mM sucrose mixed with 3 mM PAP. (*n* = 12–13). (C and D) Feeding preference (PI, preference index) of *Obp49a*^1^*/Ob49a*^1^; *+/+* (*Obp49a*) and *Gr64f-Gal4/+*; *UAS-GABA-B-R2 RNAi/+* flies choosing between 200 mM sucrose and 200 mM sucrose mixed with 2 mM PAP. (*n* = 8–15). (E) Feeding preference (PI, preference index) of *UAS-Kir2*.*1/tub-Gal80*^*TS*^; *Gr33a-Gal4/+* flies with indicated temperature shifts for larval or adult-specific silencing of *Gr33a* GRNs (*n* = 11). (F and G) Results of single sensillum tip recordings from (F) the S6 sensillum (*n* = 6–9 recordings, 4–6 insects) or (G) selected L sensilla (*n* = 19–26 recordings, 5–10 insects) stimulated with the indicated stimuli prepared in 30 mM tricholine citrate. For experiments in (A−E), flies were given control diet (naive) or control diet with 1 mM PAP (PAP-exposed) as larvae. All data represent mean ± SEM. Binary choice assays (A–E) were analyzed via Mann-Whitney test. Single sensillum responses (F and G) were analyzed via two-way ANOVA with Šídák’s multiple-comparisons test (F) or Dunnett’s T3 for pairwise comparisons (G). Data in (G) were analyzed after performing a square-root transformation after applying a Box-Cox test. ∗*p* < 0.05 and ∗∗*p* < 0.01.

To identify taste pathways involved in experience-induced alterations in feeding behavior, we examined the roles of bitter taste activation and sweet taste suppression, both of which are engaged by various bitter tastants.^33^^,^^40^^,^^41^ Gr33a is broadly expressed in bitter GRNs and functions in both larvae and adults to facilitate sensing of bitter compounds,^41^^,^^42^^,^^43^^,^^44^^,^^45^ and *Gr33a* mutants showed reduced bitter GRN responses to PAP.^42^ We therefore asked whether these flies, with disrupted PAP sensing, still exhibited modified behavioral sensitivity. In choice assays, naive *Gr33a* mutants still showed robust PAP avoidance in our experimental conditions, consistent with partial disruption of bitter sensing.^46^ However, PAP-exposed *Gr33a* mutants were no longer capable of attenuating their avoidance (Figure 6A).

Because naive *Gr33a* mutants retained substantial PAP aversion, we next considered the involvement of other co-receptors. In mutants for two other broadly expressed bitter co-receptors, Gr32a and Gr66a, weak PAP avoidance was observed independent of bitter rearing, and PAP rearing did not produce attenuation of avoidance in either genotype (Figure 6A). We then performed neuron silencing experiments using *Gr33a-Gal4* to drive expression of the inward rectifying potassium channel Kir2.1. When faced with a choice between 200 mM sucrose and 200 mM sucrose + 2 mM PAP, naive *Gr33a*-silenced flies demonstrated only weak aversion to the sucrose/PAP mixture (Figure S1A). Increasing the PAP concentration to 3 mM elicited robust aversion in naive flies, but larval exposure to PAP had no effect on choice behavior in this background (Figure 6B). Together, our results indicate that exposure-induced shifts in behavioral responses to PAP depend on normal bitter GRN activity.

Bitter tastants also influence the gustatory system through inhibition of sweet-sensing GRNs.^47^^,^^48^^,^^49^ Obp49a, a member of the odorant binding protein (Obp) family expressed in accessory cells of taste organs, facilitates sweet GRN inhibition possibly by conveying bitter chemicals to sweet taste receptors^50^; notably, this mechanism of sweet inhibition does not require bitter GRN activation. In PER assays, *Obp49a* mutants exhibited robust responses to a range of PAP concentrations in a sucrose background, consistent with disrupted sweet inhibition (Figure S1B). In our choice paradigm using sugar/bitter mixtures, *Obp49a* mutants failed to show attenuated behavioral responses following larval exposure (Figure 6C). In addition to direct sweet GRN inhibition, bitter tastants dampen sweet GRN activity through presynaptic GABAergic inhibition.^51^ GABA-B receptors, expressed in sweet *Gr64f*^+^ GRNs but not in bitter GRNs, receive inhibitory input from GABA interneurons following bitter GRN activation. We knocked down GABA-B receptor expression in *Gr64f*^+^ GRNs and found that rearing larvae on PAP-containing food no longer generated reduced adult PAP avoidance (Figure 6D). Together, these results suggest that larval exposure-dependent shifts depend on the normal function of multiple bitter-mediated pathways: activation of bitter GRNs, direct inhibition of sweet GRNs via Obp49a, and indirect GABA-mediated sweet GRN inhibition.

Our rearing paradigm exposes animals to bitter chemicals during larval development. We next asked when bitter GRN activity is required for the sensitivity shift. Using the TARGET (temporal and regional gene expression targeting) system, in which a ubiquitously expressed, temperature-sensitive GAL80 protein represses GAL4 at 18°C but not at 29°C when it is inactivated,^52^ we expressed Kir2.1 in *Gr33a*^+^ GRNs in a temporally restricted manner. Flies in which bitter GRNs were silenced during larval development failed to attenuate their adult PAP avoidance (Figure 6E, left). In contrast, flies with intact larval bitter GRN activity exhibited the expected reduction in adult PAP avoidance, despite the silencing of bitter GRNs during adulthood (Figure 6E, right). Together, these findings suggest that bitter sensing during larval development is required for experience-dependent modification of adult responses to the same bitter cues.

Having established that both bitter-sensing and sweet-inhibition pathways are required for the attenuating effects of larval PAP exposure, we next asked how this exposure modulates the physiological responses of adult taste neurons. Using single-sensillum tip recordings, we measured labellar responses from the S6 sensillum (representing S-type sensilla containing a broadly tuned bitter GRN) and selected L-type sensilla (L3–4 and L7–9), representing sensilla that lack bitter GRNs). PAP rearing did not alter S6 sensillum responses to either 1 mM or 2 mM PAP (Figure 6F). For L-type sensilla, we measured responses to 100 mM sucrose alone or in mixtures with varying concentrations of PAP. Overall, we found that larval PAP exposure had a small but significant effect on L sensillum responses to the sucrose/PAP mixtures (Figure 6G). In naive flies, responses to 100 mM sucrose + 2 mM PAP were significantly reduced relative to sucrose alone, whereas no sucrose/PAP mixture significantly reduced responses in PAP-exposed flies. It is possible that L sensillum responses are not uniformly affected, since we found no differences in sweet taste suppression between naive and PAP-exposed flies if we analyzed only L7–L9 sensilla. These results raise the possibility that larval PAP exposure may reduce PAP-mediated suppression of sweet taste in some adult GRNs.

### Bitter projection neurons and cAMP-dependent memory pathways shape experience-dependent attenuation

**Figure 7:**
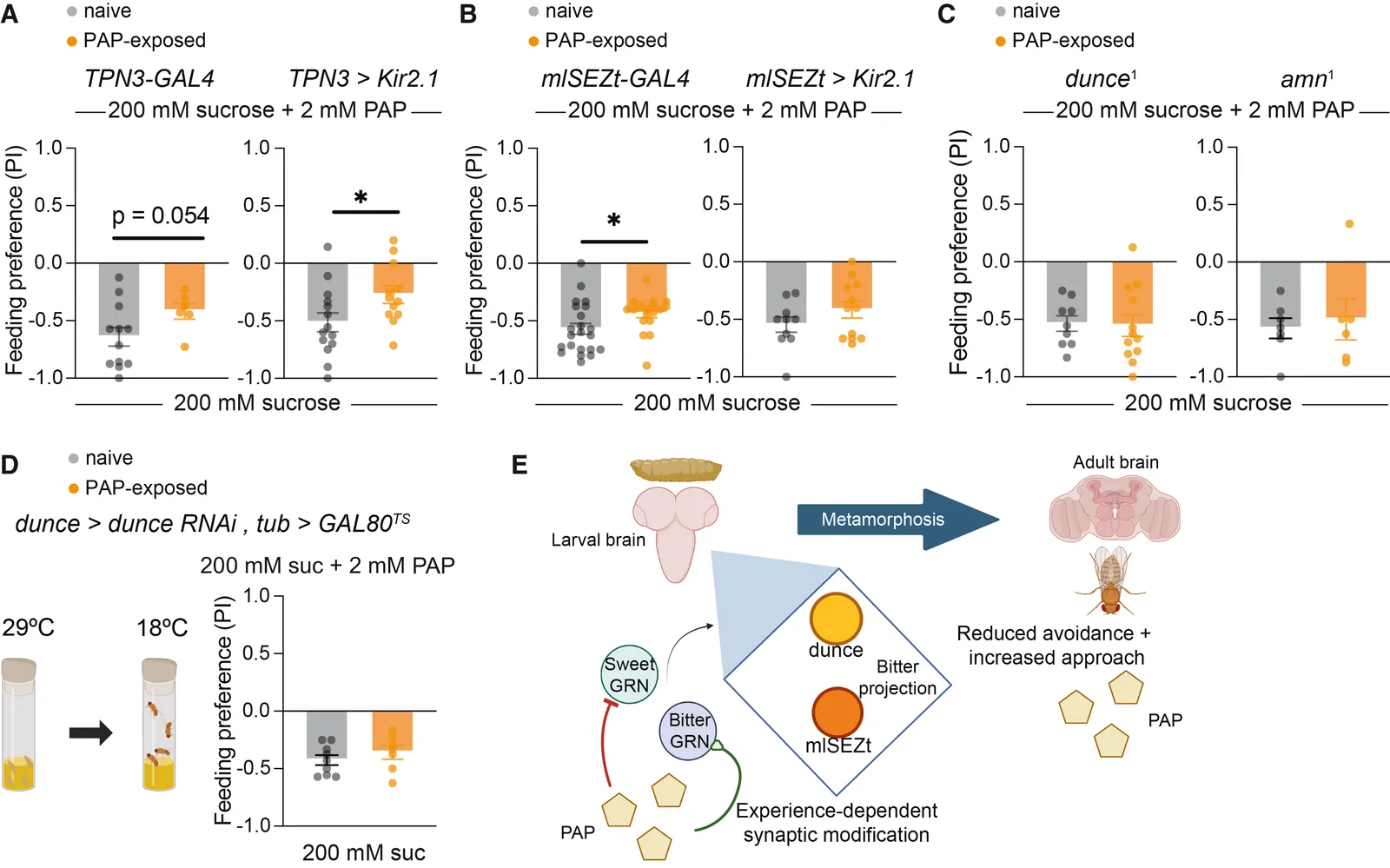
Experience-dependent PAP attenuation requires functional bitter projections and cAMP-associated memory signaling (A–C) Feeding preference (PI, preference index) of *+/+*; *R11H09-GAL4/+* (*TPN3-GAL4*), *UAS-Kir2*.*1/+*; *R11H09-GAL4/+* (*TPN3 > Kir2*.*1*), *+/+* (A); *R29F12-GAL4/+* (*mlSEZt-GAL4*), *UAS-Kir2*.*1/+*; *R29F12-GAL4/+* (*mlSEZt > Kir2*.*1*) (B), *dnc*^1^*/dnc*^1^; *+/+*; *+/+* (*dunce*), and *amn*^1^*/amn*^1^; *+/+*; *+/+* (*amn*) (C) flies choosing between 200 mM sucrose and 200 mM sucrose mixed with 2 mM papaverine (PAP). (*n* = 6–12). (D) Feeding preference (PI, preference index) of *dnc-GAL4/+*; *UAS-dncRNAi/+*; *tub-GAL80*^*TS*^*/+* flies with indicated temperature shift for larval-specific *dunceRNAi* using GAL80^TS^ (*n* = 8–10). (E) A model for larval experience-dependent desensitization. PAP suppresses sweet taste and is detected by bitter GRNs, which inhibit sweet taste processing. Larval exposure may trigger synaptic modifications requiring the *dunce* pathway and mlSEZt^+^ projection neuron activity, leading to reduced avoidance in adults. For experiments in (A–D), flies were given control diet (naive) or control diet with 1 mM PAP (PAP-exposed) as larvae. Bars represent mean ± SEM. All panels analyzed via Mann-Whitney tests. ∗*p* < 0.05.

Taste information is integrated in the brain to shape future food interactions and to optimize metabolic homeostasis and survival. An open question, then, is which neurons convey larval taste information to influence adult behavioral outcomes. Previous studies have identified two functional bitter projection neuron populations, TPN3 and mlSEZt.^53^^,^^54^ Data from the Janelia FlyLight collection indicate that only mlSEZt neurons are present in both larval and adult stages.^55^^,^^56^ Consistent with the absence of a functional role for TPN3 in larval bitter signaling, silencing TPN3 neurons did not affect the consequences of larval PAP exposure (Figure 7A). Silencing mlSEZt neurons, in contrast, prevented the reduced-avoidance phenotype that PAP-exposed flies otherwise exhibited (Figure 7B), suggesting that mlSEZt activity during larval development contributes to behavioral desensitization.

Since mlSEZt neurons project to higher order brain regions such as the superior lateral protocerebrum (SLP) and lateral horn (LH), taste information conveyed by these neurons could lead to synaptic modifications in these regions that influence future feeding decisions. Such synaptic changes are likely to involve molecular mechanisms associated with learning and memory. We therefore examined the roles of two memory-related genes, *dunce* and *amnesiac*, in mediating larval influences on adult feeding behavior. *dunce* mutants, deficient in the phosphodiesterase that degrades cAMP during synaptic modification, were unable to modify their adult response following larval PAP exposure (Figure 7C). *amnesiac* mutants, impaired in neuropeptide synthesis that controls cAMP production, similarly failed to show larval experience-dependent behavioral changes in the adult (Figure 7C). These findings are consistent with, although do not directly demonstrate, a role for plasticity downstream of taste neurons in encoding the effect of experience.

We next asked whether memory-related gene expression is required during larval development, when flies first encounter the altered dietary condition. Using the TARGET system we selectively knocked down *dunce* expression in larvae and found that flies were no longer able to attenuate their adult PAP avoidance (Figure 7D), indicating that intact memory consolidation pathways are required in larvae for developmental experience-dependent shifts in adult behavior.

## Discussion

We report pathways through which larval taste sensing contributes to modified feeding behaviors in response to a specific bitter compound across metamorphosis. Our study characterizes and validates a tastant-exposure model for functional dissection of pre-imaginal modulation of taste responses in adult *Drosophila*. Behavioral, physiological, and metabolic analyses revealed that larval exposure to PAP, a bitter isoquinoline alkaloid, was sufficient to attenuate innate avoidant responses through mechanisms involving bitter taste sensing and memory-related pathways.

Our experiments targeting *Gr33a*^+^ GRN function, mlSEZt neuron populations, and *dunce* expression collectively implicate a circuit that connects peripheral, PAP-sensing *Gr33a*^+^ bitter GRNs with downstream processing of this experience. The downstream processing appears to require mlSEZt projection neuron activity and dunce-mediated consolidation (Figure 7E). Because the genetic tools we used affect broad populations of neurons, further investigation will be required to identify specific roles in taste memory versus other processes.

The compound specificity of our paradigm raises important mechanistic questions. We found that larval exposure to PAP, but not DEN or NOS, elicited shifts in adult behavioral responses to the same compound, and that larval exposure to NOS or DEN appeared to enhance rather than reduce adult PAP avoidance. Several hypotheses merit consideration. First, the tested concentration range may not have captured the dynamic range optimal for DEN-induced behavioral shifts, although in naive flies the concentrations we used elicited dose-dependent effects comparable to those of PAP. Second, different bitter compounds likely recruit distinct receptors and GRNs both within and across larval taste organs,^57^ which could explain compound-specific plasticity; bitter discrimination may also occur through temporal coding of excitatory bitter neuron responses.^58^ Third, the asymmetric effects of cross-compound exposure could reflect interactions between bitter-sensing pathways or differential recruitment of inhibitory mechanisms. Identifying specific tuning receptors and neurons that distinguish PAP sensing from the other tastants in our paradigm may permit further examination of the phenotype’s ligand specificity. The observation that this plasticity appears tuned to the identity of the chemical encountered during development could point to a logic that allows flies to adapt to specific persistent toxins without compromising overall protective avoidance behaviors. In addition, early-life environmental stressors, including nutritional restriction and oxidative stress, are known to produce persistent changes in adult sensory physiology and behavior.^59^^,^^60^ Although speculative, such mechanisms highlight additional routes by which compound-specific or cross-compound exposure could lead to asymmetric effects.

The sex-specific differences we observed warrant careful interpretation. PAP exposure effects were most pronounced in females, while CAF exposure effects were male-specific. Labellar taste pegs and tarsal taste hairs show sexual dimorphism in sensillum numbers and receptor expression.^61^^,^^62^^,^^63^ However, whether these anatomical differences account for the observed behavioral dimorphism remains unclear. Alternative explanations include sex-specific differences in metabolic sensitivity to alkaloids, hormonal influences on taste sensitivity, or sex-specific developmental timing effects. The molecular basis for these differences represents an important area for future investigation.

Several mechanisms could account for the experience-dependent modulation of feeding behavior. Although it has been shown that bitter-sensing GRNs become desensitized following both long- and short-term exposure in adults,^64^^,^^65^ PAP-mediated bitter neuron response in S6 sensilla seems to be unaffected by larval PAP rearing (Figure 6F). While it remains possible that other bitter GRNs are modulated by larval exposure, the observation is consistent with the finding that adult bitter GRN activity appears to be dispensable for the behavioral shift. Moreover, bitter neuron activity is required during larval rearing suggesting that while rearing may not alter bitter neuron responsiveness itself, bitter sensory input during development is required for the later behavioral modification. GRN desensitization is unlikely to occur through changes in *Gr33a*, because we did not observe generalization to other *Gr33a*-dependent bitter tastants. However, *Gr33a* also labels pharyngeal GRNs,^66^ which remain intact throughout metamorphosis, and because our conditional silencing of *Gr33a*^+^ neurons targets both external and internal populations, the observed behavioral desensitization may result from changes in the pharynx as well. Future studies will be required to decouple the relative contributions of pharyngeal versus labellar input in shifting feeding preference. Interestingly, L-type sensillar recordings raise the possibility that sweet taste suppression may be modulated upon larval PAP rearing (Figure 6G). While the contribution of different sensillar classes must be dissected in future studies, the results suggest that developmental exposure may directly or indirectly dysregulate mechanisms underlying bitter tastant-mediated suppression of sweet neuron activity.^67^

Second, central controllers of avoidance pathways might habituate with repeated exposure, consistent with our findings implicating *dunce* and mlSEZt neurons. Third, classical reward conditioning could result from pairing bitter stimuli with the nutritive properties of larval food, creating positive associations that persist into adulthood. Fourth, epigenetic modifications could create a molecular memory that survives metamorphosis, as suggested by recent work demonstrating diet-induced chromatin changes that affect behavior.^68^^,^^69^

The challenge of trans-metamorphic behavioral persistence remains a central puzzle. Most larval taste neurons do not survive metamorphosis,^25^ and extensive mushroom body reorganization occurs during this period.^12^ However, several mechanisms could enable information transfer. Pharyngeal taste neurons, many of which survive metamorphosis, represent potential anatomical substrates for taste memory. Alternatively, larval taste experiences could induce epigenetic modifications that influence adult circuit development, creating anatomical or functional distinctions in neuronal or accessory components of taste pathways. Recent evidence for trans-metamorphic persistence of memory circuits through synaptic remodeling^12^ provides a framework for understanding how larval experiences could influence adult behavior without requiring direct neural continuity.

Ecological considerations support the biological relevance of developmental taste plasticity. In natural environments, developing insects encounter diverse plant secondary compounds, including alkaloids, with concentrations that vary across host plants and seasons. The ability to adjust behavioral responses based on early exposure could provide adaptive advantages in environments where resource quality fluctuates. It will therefore be important to test ecologically relevant concentrations of plant-derived tastants, ideally in the context of natural complex stimuli, to further probe the ecological relevance of larval-experience-induced shifts in adult behavior.

In conclusion, our work establishes a model for investigating how early taste experiences influence adult behavior in *Drosophila*. The compound-specific effects we observed suggest sophisticated mechanisms underlying developmental taste plasticity, while the involvement of memory-related pathways indicates potential conservation with other forms of experience-dependent behavioral modification. Our study lays the foundation for future work to understand the mechanisms underlying developmental taste plasticity and its broader implications for behavioral programming across metamorphosis.

### Limitations of the study

Our work provides key insights into behavioral modification of taste behavior following developmental exposure, but several limitations must be acknowledged. First, our conclusions rely predominantly on behavioral assays, complemented by some peripheral physiological recordings. Second, although our study focused on three bitter compounds, the specificity of effects will require validation across a broader panel of taste stimuli that spans multiple chemical classes. Third, we did not examine molecular-level changes such as gene expression or chromatin modifications resulting from chronic exposure, which leaves open questions regarding mechanistic pathways. Fourth, alternative explanations should be considered: the behavioral changes we report could reflect non-specific effects of early chemical exposure rather than specific taste plasticity. For example, mild developmental stress could induce compensatory changes in feeding behavior that manifest as apparent taste modifications, and the cross-compound effects we observed could reflect interactions between developing taste pathways rather than specific plasticity mechanisms. Despite these limitations, our findings provide a foundation for future research into developmental plasticity of sensory behavior and its broader implications for understanding how early experiences shape adult behavioral repertoires.

## Resource availability

### Lead contact

Further information and requests for resources and reagents should be directed to and will be fulfilled by the lead contact, Anupama Dahanukar (anupama.dahanukar@ucr.edu).

### Materials availability

This study did not generate new unique reagents.

### Data and code availability

• All original data have been deposited at Mendeley Data and are publicly available at Mendeley Data: https://doi.org/10.17632/7sxh6df8pr.1. Fly stock and chemical identifiers are included in the key resources table.
• The paper does not report original code.
• Any additional information required to reanalyze the data reported in this paper is available from the lead contact upon request.

## Acknowledgments

We thank members of the Dahanukar and Ray lab for feedback. Additional thanks to S. Haga-Yamanaka, K. Abdulrazak, Y.C. Chen, M. Dey, and A. Ganguly for helpful discussions. This work was supported by funds from the 10.13039/100005595University of California, Riverside Agricultural Experimental Station, 10.13039/100005825National Institute of Food and Agriculture–10.13039/100000199U.S. Department of Agriculture (Hatch Project grant no. 1011543), the Department of Education’s Graduate Assistance in Areas of National Need award, and the 10.13039/100007602University of California, Riverside (Dissertation Completion Fellowship Award).

## Author contributions

Conceptualization, V.D.M. and A.D.; validation, V.D.M.; formal analysis, V.D.M.; investigation, V.D.M., C.C., M.V.-M., and A.D.; writing – original draft, V.D.M. and A.D.; writing – review and editing, V.D.M., C.C., and A.D.; visualization, V.D.M.; supervision, A.D.; funding acquisition, V.D.M. and A.D.

## Declaration of interests

A.D.’s spouse is a founder and president of two startups, Sensorygen Inc. and Remote Epigenetics Inc., and is listed as inventor on multiple patents filed by UC Riverside.

## Declaration of generative AI and AI-assisted technologies in the writing process

During the preparation of this work, the authors used Claude and ChatGPT 5.5 to improve readability and verify consistency between responses to reviewers and corresponding revisions in the manuscript text. The authors reviewed and validated all manuscript content, interpretations, and conclusions. No AI tools were used for data analysis, scientific conclusions, or figure generation. After using these tools, the authors reviewed and edited the manuscript and take full responsibility for the content of the publication.

## STAR★Methods

### Key resources table

**Table:** 

REAGENT or RESOURCE	SOURCE	IDENTIFIER
**Chemicals, peptides, and recombinant proteins**		

Sucrose	Sigma-Aldrich	S7903
Caffeine	Sigma-Aldrich	C8960
Papaverine hydrochloride	Sigma-Aldrich	P3510
Denatonium benzoate	Sigma-Aldrich	D5765
Noscapine hydrochloride	Sigma-Aldrich	N9007
Indigo carmine	Sigma-Aldrich	I8130
Sulforhodamine B	Sigma-Aldrich	230162
Tricholine citrate	Sigma-Aldrich	T0252
Methanol	Sigma-Aldrich	34860

**Deposited data**		

Raw data	Mendeley Data	Mendeley Data: https://doi.org/10.17632/7sxh6df8pr.1

**Experimental models: Organisms/strains**		

*D. melanogaster*: CantonS	John Carlson	FlyBase: FBsn0000274
*D*. *melanogaster*: *Gr33a*^1^	Bloomington *Drosophila* Stock Center	BDSC:31427; FlyBase: FBti0168370
*D*. *melanogaster*: *Gr32a*^1^	Hany Dweck	FlyBase: FBti0146019
*D*. *melanogaster*: *Gr66a*^1^	Hany Dweck	FlyBase: FBal0358297
*D*. *melanogaster*: *Gr33a-GAL4*	Bloomington *Drosophila* Stock Center	BDSC:57624; FlyBase: FBti0162706
*D*. *melanogaster*: *UAS-Kir2*.*1*	Bloomington *Drosophila* Stock Center	BDSC:6596; FlyBase: FBti0017551
*D*. *melanogaster*: *Obp49a*^1^	Bloomington *Drosophila* Stock Center	BDSC:55033; FlyBase: FBti0168618
*D*. *melanogaster*: *UAS-GABA-B-R2 RNAi*	Bloomington *Drosophila* Stock Center	BDSC:27699; FlyBase: FBti0128881
*D*. *melanogaster*: *tubP-GAL80*^TS^	Bloomington *Drosophila* Stock Center	BDSC:7019; FlyBase: FBti0128881
*D*. *melanogaster*: *dnc*^1^	Bloomington *Drosophila* Stock Center	BDSC:6020; FlyBase: FBal0002713
*D*. *melanogaster*: *amn*^1^	Bloomington *Drosophila* Stock Center	BDSC:5954; FlyBase: FBal0000500
*D*. *melanogaster*: *UAS-dnc RNAi*	Bloomington *Drosophila* Stock Center	BDSC:78786; FlyBase: FBti0199263
*D*. *melanogaster*: *R11H09-GAL4 (TPN3-GAL4)*	Bloomington *Drosophila* Stock Center	BDSC:48478; FlyBase: FBti0133011
*D*. *melanogaster*: *R29F12-GAL4 (mlSEZt-GAL4)*	Bloomington *Drosophila* Stock Center	BDSC:49495; FlyBase: FBti0134634

**Software and algorithms**		

Prism 11	Graphpad	RRID:SCR_002798
Illustrator	Adobe	RRID:SCR_010279
BioRender	BioRender	RRID:SCR_018361
Autospike	Ockenfels-Syntech	N/A

### Experimental model and study participant details

#### Fly stocks

Naïve flies were raised on standard cornmeal-glucose-yeast media. Larval exposure models required rearing flies on standard food containing 1 mM of the specified bitter tastant. Flies were reared at 25°C in a 12/12 light-dark cycle, unless otherwise indicated. For wild-type experiments, we used Canton-S. The following other mutant and transgenic stocks were used – *Gr33a*,^1^ *Gr32a*,^1^ *Gr66a*,^1^ *Gr33a-GAL4*, *UAS-Kir2*.*1*, *Obp49a*,^1^ *UAS-GABA-B-R2 RNAi*, *tub-GAL80*^ts^, *dunce*,^1^ *amnesiac*,^1^ *UAS-dunce RNAi*, *R11H09-GAL4* (*TPN3-GAL4*), and *R29F12-GAL4* (*mlSEZt-GAL4*). *Gr32a*^1^ and *Gr66a*^1^ mutants were obtained from Dr. Hany Dweck; all other stocks were acquired from the Bloomington *Drosophila* Stock Center (BDSC).

### Method details

#### Fly rearing

Treatment food media was made by heating standard media until it was resuspended. Bitter tastant solutions were added until the final concentration of bitter in food was achieved (1 mM for all tastants used in exposure paradigm). Flies added to treated food and allowed to seed bottles. Embryo were allowed to develop. Once they reached late third instar (marked by “wandering” phenotype, where they emerge from food to prepare for pupation) or early pupation, flies were collected from treatment vials. They were rinsed with deionized water on a sieve made by perforating a sheet of aluminum foil with a 25-gauge hypodermic needle. Larvae and pupae were rinsed and gently agitated with a paintbrush until water ran clear and no food debris visibly remained. Larvae and pupae were then transferred to fresh vials containing standard food, where they were allowed to eclose. To account for possible effects of handling and rinsing, flies reared in standard food as larvae were treated to the same collection, wash, and transfer protocol with separate sieves and paintbrushes.

#### Pupariation rate assay

Mated adult Canton-S flies were allowed lay eggs on a 0.75% agarose 200 mM sucrose substrate overnight. On the following day, a paintbrush was used to seed vials containing standard fly food laced with one of 1 mM papaverine hydrochloride, 1 mM denatonium benzoate, or 1 mM caffeine with 10 unhatched eggs. Vials were placed in an incubator at 25°C and 50% humidity and monitored each day to count the total number of puparia that formed in each vial.

#### Eclosion rate assay

Experiments were set up in same manner as the pupariation rate assay, with additional steps. After all pupae were formed, total number pupae in each vial were counted, and vials were monitored each day to count the total number of adult flies that emerged from each vial. Number of adults were normalized to the starting number of pupae in each vial.

#### Developmental timing assay

Experiments were set up in the same manner as the eclosion rate assay, with additional steps. Total number of adults in each vial were counted on each day as they emerged. Flies first began to emerge 10 days after seeding and continued emerging until 12 days post seeding. For each vial, an average development time value was calculated by multiplying the day of emergence by the number of emerged adults on that day and dividing by the total number of emerged flies.

#### Larval consumption assay

Mated adult Canton-S flies were allowed to lay eggs in vials filled with standard food or food laced with a bitter substance (1 mM PAP, CAF, or DEN) for 24 hours. Adults were then removed and the vials were placed in an incubator at 25°C and 50% humidity. Approximately 4 days after egg laying, L3 larvae were floated out of food using a 20% sucrose solution and placed into a 60 x 15 mm petri dish filled with either standard food or food laced with a bitter substance and allowed to feed for 1 hour. Food used in the assay was dyed red using Satin Ice Red Rouge Gel food coloring (0.75%) to track rates of consumption. After 1 hour of feeding, larvae were floated out of petri dishes with a 20% sucrose solution and placed into a 1.5 mL microcentrifuge tube. Plates with less than 10 larvae recovered were excluded from analysis. Tubes were snap frozen in liquid nitrogen and larvae were homogenized into a fine powder using a pestle. 15 uL of a methanol/water mixture was added to each tube after complete homogenization and larval debris was spun down. Absorption of the supernatant was measured at 520 nm using a Nanodrop 2000c Spectrometer. A standard curve was generated for the dye using known concentrations to determine amount of food consumed per each group of ten larvae.

#### Binary choice feeding assays

Feeding preference assays were performed as described previously,^70^ with slight modifications. Unless otherwise specified, *Drosophila* aged 2-4 days post-eclosion were used for experiments. 24 hours prior to the experiment, 16-20 flies (8-10 females + 8-10 males) were starved in vials containing soaked Kimwipes to prevent dehydration. 10uL droplets of sucrose or sucrose/bitter food dissolved in a 0.75% agarose medium were added to tight-fit petri dishes (Falcon 35-1006). Each food choice was dyed either with indigo carmine (Sigma, #I8130) (0.25mg/mL) or sulforhodamine B (0.5mg/mL) (Sigma, #230162). To account for potential dye-preference interactions, we performed dye swaps except for PAP. Since we observed that papaverine-laced food diminished visibility of indigo carmine over time, all experiments with papaverine were performed with indigo carmine for sucrose and sulforhodamine B for sucrose + papaverine. Flies were allowed to feed in the plates for 2 hours at 25°C and 50% humidity in a dark container. Following this, plates were frozen, and abdomens were dissected for visual scoring, with flies grouped by sex. Trials in which at least 50% of all flies and at least 5 flies of each sex participated were included. Preference index for each group of males or females within a plate was calculated using: [(# flies with sucrose/bitter) – (# flies with sucrose)] / (total # flies).

#### Residence assay

Flies were starved for 24 hours in vials containing water-soaked Kimwipes prior to testing. Binary choice dishes were made using 35 mm Petri plates following a previously described protocol.^71^ 8-12 female flies were briefly anesthetized on ice and gently placed into plates using forceps. The number of flies contacting each substrate was counted every 15 minutes for 2 hours. To normalize for differences in total number of flies on each plate, the total number of “spottings”– instances where a fly contacted a given food substrate – per plate was divided by the number of flies.

#### Proboscis extension reflex (PER) assay

Flies were starved for 24 hours in vials containing water-soaked Kimwipes prior to testing. For labellar-evoked PERs, flies were aspirated into a pre-cut P200 pipette tip so that the fly head was exposed while securing the thorax and abdomen. The tip containing the fly was then mounted on a microscope slide using clay. Stimuli were presented using approximately 1 mm thin strips of Kimwipes twisted into a fine twine and then dipped into tastant solutions. For tarsal-evoked PERs, flies were gently fixed to the surface of a microscope slide by placing droplets of nail polish to the slide and affixing flies to the droplet by their dorsal side. Flies were allowed to recover from fixation to the slide for at least 30 minutes. Stimuli were presented using P10 pipette tips.

In both experiments, flies were first offered water to satiety to ensure extensions were not due to dehydration. They were then tested with each chemical in tastant panel three times. The order of tastants was blinded to the experimenter and shuffled before each experimental session. Flies that did not respond positively to positive control 200 mM sucrose were excluded from analysis, as were flies that continued to extend in response to consecutive water presentations. For each eligible fly, response rate to each tastant was calculated by dividing the # of positive extensions (either 0, 1, 2, or 3) by 3. Flies that did not respond to 200 mM sucrose or exhibited sustained or consecutive positive responses to water were excluded from analysis.

#### FLIC assay

Fly Liquid Interaction Counter (FLIC) assays were performed either in a single-food or binary choice arena. Data were collected from three Drosophila Feeding Monitor (DFM) plates.^38^ Mated female flies were starved for 24 hours in vials containing soaked Kimwipes. 2-4 day old flies were assayed for 1 hour. The first 5 minutes were excluded from analysis to accommodate loading flies into DFMs. FLIC data were analyzed using scripts from the Pletcher Lab.

#### Mass spectrometry

All mass spectrometry experiments were performed at the UC Riverside Metabolomics Core Facility. Metabolite analysis was performed on a TQ-XS triple quadrupole mass spectrometer (MS) (Waters) coupled to an I-class UPLC system (Waters). Separations were carried out on a ACQUITY UPLC BEH C18 column (2.1 x 100 mm, 1.7 μM) (Waters). The mobile phases were (A) water with 0.1% formic acid and (B) acetonitrile with 0.1% formic acid. The flow rate was 250 μL/min, and the column was held at 40°C. The injection volume was 1 μL. The gradient was as follows: 0 min, 98% A; 0.1 min, 98% A; 2.5 min, 2% A; 8 min, 2% A; 8.1 min, 98% A; 12 min, 98% A. The retention time of the metabolite was 2.93 minutes.

The MS was operated in multiple reaction monitoring mode. Transitions 202.10 and 171.04 from parent mass 340.30 were detected. Source and desolvation temperatures were 150°C and 600°C, respectively. Desolvation gas was set to 600 L/hr and cone gas to 150 L/hr. Collision gas was set to 0.15 mL/min. All gases were nitrogen except the collision gas, which was argon. The capillary voltage was 1 kV in positive ion mode. System stability was monitored by analyzing a quality control sample (generated by pooling together equal volumes of all sample extracts) every six injections. Samples were analyzed in random order.

#### Extracellular recordings

Extracellular tip recordings from adult labellar taste sensilla were performed as previously described using a Syntech TastePROBE and IDAC4 data acquisition system with Autospike software.^72^ Briefly, flies were immobilized and a glass reference electrode filled with Ringer solution was inserted through the thorax and proboscis into the labellum. Taste stimuli were dissolved in 30 mM tricholine citrate and applied to the tips of individual sensilla using a glass recording electrode. Recordings were obtained from selected L-type sensilla and from the S6 sensillum, which was used as the representative S-type sensillum for bitter neuron responses. Each stimulus contact lasted approximately 2–3 s, and applications of different stimuli to the same sensillum were separated by > 1 min. Neural responses were quantified by counting action potentials for 500 ms upon onset of the response and converted to spikes per second.

For L-type sensilla recordings, spike counts were square-root transformed prior to statistical analysis. This transformation was selected based on Box-Cox analysis of the L-type sensilla dataset, which estimated a λ value of approximately 0.67 with a confidence interval that did not include 1 but included 0.5, supporting use of a square-root transformation. The same analysis did not support transformation of the S-type sensilla dataset, for which the confidence interval included 1, indicating that transformation was not warranted.

### Quantification and statistical analysis

For each experiment, we first analyzed the distribution of data using the Kolmogorov-Smirnov test. If the distribution was normal, we proceeded with Welch’s *t-*tests or Mann-Whitney U test for experiments with two groups, unless otherwise specified. For multidimensional data sets (where variables were rearing condition and concentration), 2-way ANOVAs were performed, followed by *post hoc* Sidak’s pairwise comparisons with corrections to compare rearing conditions at each dosage. Sample sizes for each experiment are reported in the corresponding figure legends, and exact statistical tests, test statistics, and *p*-values for every figure panel are tabulated in Table S1.

## Supplemental information

Document S1. Figure S1

Table S1. Summary of statistical analyses
