# Supplementary material for: Larval taste exposure creates compound-specific behavioral modifications in adult Drosophila melanogaster
Source: iScience. 2026 Sep 8;29(9):117467. doi: 10.1016/j.isci.2026.117467 (PMC13573713; doi:10.1016/j.isci.2026.117467)
Supplement: Supplementary file 1 [file mmc1.pdf]

iScience, Volume *29*

Supplemental information

Larval taste exposure creates compound-specific behavioral modifications in adult *Drosophila melanogaster*

Vaibhav D. Menon, Christopher Creighton, Mayra Vazquez-Muñoz, and Anupama Dahanukar

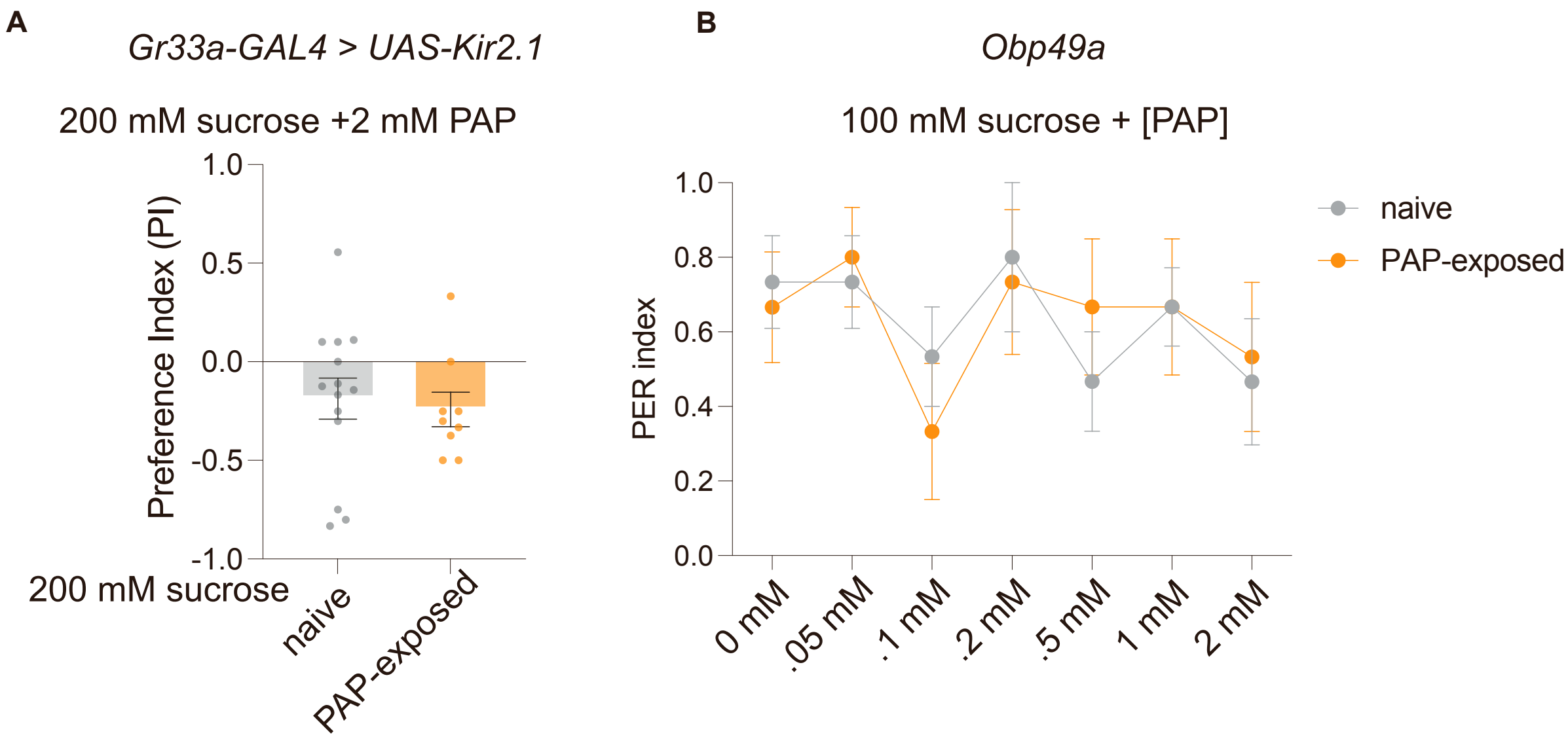

Figure S1, related to Figure 6. Bitter silencing and disruption of sweet neuron inhibition reduces PAP sensitivity.

(A) Preference Index (PI) comparing naïve and PAP-exposed adult *Gr33a-GAL4>UAS-Kir2.1* females (n = 9-14). **(B)** PER index for female *Obp49a* adults offered 100mM sucrose supplemented with increasing concentrations of (PAP) via labellar stimulation (n = 5 flies). Bars and points represent mean ± SEM. Individual points in preferenc assay data represent separate trials. Binary choice assay **(A)** analyzed via Mann-Whitney test. PER assay **(B)** analyzed via two-way ANOVA, followed by Šídák's multiple comparisons test.
